# Updates in the Use of BCL-2-Family Small Molecule Inhibitors for the Treatment of Relapsed/Refractory Multiple Myeloma

**DOI:** 10.3390/cancers14143330

**Published:** 2022-07-08

**Authors:** Ricardo D. Parrondo, Aneel Paulus, Sikander Ailawadhi

**Affiliations:** 1 Division of Hematology-Oncology, Mayo Clinic, Jacksonville, FL 32224, USA; parrondo.ricardo@mayo.edu; 2 Department of Cancer Biology, Mayo Clinic, Jacksonville, FL 32224, USA; paulus.aneel@mayo.edu

**Keywords:** multiple myeloma, Bcl-2 inhibitors, apoptosis, small molecule inhibitors

## Abstract

**Simple Summary:**

Monumental therapeutic advances have been made over the past two decades for the treatment of multiple myeloma. Anti-apoptotic proteins, such as Bcl-2, Bcl-xL, and Mcl-1, have been found to be upregulated in multiple myeloma cell lines, and small molecule inhibitors that target these proteins are in clinical development with the goal of enhancing apoptosis, reversing drug resistance, and improving the survival outcomes of patients with relapsed/refractory multiple myeloma. In this paper, we review the available clinical data for the Bcl-2-family protein inhibitors currently in clinical development for relapsed/refractory multiple myeloma.

**Abstract:**

Despite considerable advances in the treatment of multiple myeloma over the past decade, progression of disease is inevitable, and patients ultimately succumb to relapsed and refractory disease. Efficacious therapeutic regimens that target the key biological pathways that are essential for malignant plasma cell survival are necessary in the efforts to improve patient survival outcomes. The Bcl-2 family of proteins comprise oncogenes that promote myeloma cell survival by conferring resistance to apoptosis. These proteins are frequently upregulated in myeloma cells, thus making them attractive therapeutic targets. Several small molecule inhibitors of Bcl-2-family proteins are currently in clinical development for the treatment of relapsed/refractory multiple myeloma. Venetoclax, a Bcl-2-specific inhibitor, has generated the most clinical data and has shown promising results in patients with multiple myeloma harboring the t (11;14) translocation. Venetoclax has shown efficacy when combined with anti-CD38 monoclonal antibodies, immunomodulatory drugs, and proteasome inhibitors. Several other Bcl-2 inhibitors are in clinical development, as are inhibitors of Mcl-1, a Bcl-2-family oncoprotein that is perhaps more critical for myeloma cell survival than Bcl-2. This review will summarize the latest clinical data regarding the clinical development of Bcl-2-family protein inhibitors in the treatment of relapsed/refractory multiple myeloma.

## 1. Introduction

Multiple myeloma (MM) is a plasma cell malignancy characterized by the clonal expansion and proliferation of malignant plasma cells in the bone marrow, and the secretion of monoclonal protein in the blood and/or urine with associated end-organ damage [1]. With the introduction and continued development of novel agents such as immunomodulatory agents (IMiD), proteasome inhibitors (PI), and monoclonal antibodies (MoAb), the survival of patients with MM continues to improve over time [2,3,4]. Relapsed disease, however, is inevitable with subsequent remissions becoming progressively shorter due to the development of refractory disease [5]. Patients with triple-class refractory MM, defined as MM that is refractory to an IMiD, a PI, and an anti-CD38 MoAb, have a poor prognosis, with a median PFS of 3.4 months and a median OS of 9.3 months with subsequent theapies following withanti-CD38 MoAb failure [6]. Novel therapeutic regimens, which target the critical pathways that are detrimental to MM cell survival, are urgently needed for patients with relapsed/relapsed refractory multiple myeloma (RRMM).

**Figure 1:**
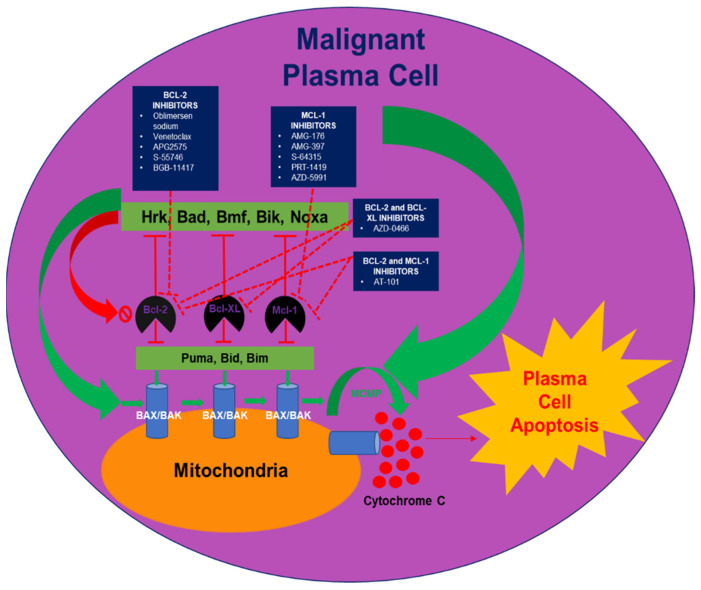
Bcl-2 family protein interactions and drug targeting inhibition of anti-apoptotic Bcl-2 family proteins. Pro-apoptotic BH3 only sensitizer proteins Hrk, Bad, Bmf, Bik, and Noxa selectively bind to and inhibit the anti-apoptotic Bcl-2 family members Bcl-2, Bcl-XL, Mcl-1 (and other proteins not depicted here). Anti-apoptotic Bcl-2 family proteins sequester BH3 only apoptotic activator proteins Puma, Bid, and Bim. Free BH3 only apoptotic activator proteins induce the oligomerization of apoptotic effectors BAK and BAX at the mitochondrial membrane which in turn cause mitochondrial outer membrane permeabilization (MOMP) and the release of cytochrome C into the cytosol ultimately resulting in plasma cell apoptosis. Oblimersen sodium, venetoclax, APG2575, S-55746 and BGB-11417 inhibit Bcl-2. AMG-176, AMG-397, S-64315, PRT-1419, and AZD-5991 are Mcl-1 inhibitors. AZD-0466 inhibits both Bcl-2 and Bcl-xL. AT-101 inhibits Bcl-2 and Mcl-1.

Malignant plasma cells exhibit many abnormalities that should normally induce cell-death signaling through the intrinsic apoptotic pathway [7]. The mitochondrial or intrinsic apoptotic pathway is regulated by the Bcl-2 family of proteins, a family of three functional protein groups, with Bax and Bak being key effectors of triggering the apoptotic cascade. Bax and Bak are kept inactivated by pro-survival Bcl-2 proteins such as Bcl-2, Mcl-1, Bcl-xL, Bcl-w, and Bcl-2A1, which act to prevent Bak and Bax activation and the subsequent initiation of the apoptotic cascade. Physiologic stresses (such as DNA damage or proteasomal stress induced by antineoplastic therapy) trigger increased expression of the BH3-only proteins Bim, Bad, and Noxa, which act to initiate apoptosis by binding tightly to pro-survival Bcl-2 proteins, which, in turn, overwhelm the capability of pro-survival proteins to inhibit Bak and Bax. This process allows Bak and Bax to oligomerize and form pores on the mitochondrial surface. Pore formation leads to mitochondrial outer-membrane permeabilization, which commits a cell towards apoptosis by allowing leakage of mitochondrial contents, such as cytochrome c, which triggers proteolytic enzymes (caspases) and culminates in the apoptosis of the cell [7]. Anti-apoptotic proteins, such as Bcl-2, Bcl-xL, and Mcl-1, have been found to be upregulated in MM cell lines, with some cells being dependent on Bcl-2-family proteins for survival [8]. This makes anti-apoptotic Bcl-2-family proteins an attractive therapeutic target for the treatment of RRMM (Figure 1). In this review, we summarize the currently available clinical data, as well as the planned clinical trials involving Bcl-2-family protein inhibitors, such as oblimersen sodium, AT-101, venetoclax, APG-2575, S-55746, BGB-11417, AZD-0466, AMG-176, AMG-397, S-64315, PRT-1419, and AZD-5991, in the treatment of RRMM (Table 1).

## 2. Oblimersen Sodium

Oblimersen sodium is a Bcl-2 antisense oligonucleotide that is complementary to the first six codons of the open-reading frame of Bcl-2 mRNA. Oblimersen sodium forms a heteroduplex that leads to the degradation of Bcl-2 mRNA, which ultimately results in the decreased translation of Bcl-2 proteins [9,10]. In a randomized phase III trial, 224 RRMM patients who had received a median of three prior lines of therapy were randomized to either dexamethasone or dexamethasone plus oblimersen sodium [11]. There were no significant differences in objective response rates between oblimersen/dexamethasone (15%) and dexamethasone alone (17%). Additionally, there was no significant difference in the time to progression for patients treated with either oblimersen/dexamethasone or dexamethasone alone. In patients who received oblimersen/dexamethasone, adverse events (AE) occurring in ≥ 25% of patients included constipation (31%), diarrhea (27%), nausea (41%), vomiting (25%), fever (48%), insomnia (35%), arthralgia (31%), and anemia (33%). Serum creatinine elevation was noted in 25% of the oblimersen/dexamethasone patients vs. 15% of the dexamethasone patients, and was ≥ Grade 3 in 8% and 4%, respectively [11]. In a phase II trial of oblimersen sodium, thalidomide, and dexamethasone in patients with RRMM, 33 patients received the triplet combination after a median of three prior lines of therapy [12]. Patients received a median of eight cycles of treatment (range: 1–16) with oblimersen sodium, thalidomide, and dexamethasone. Renal toxicity was noted, with reversible increases in serum creatinine from a median of 1.2 (0.6–2.5) at the baseline level to 1.5 (range: 0.9–2) during cycle six of therapy. For most patients, the dose of oblimersen sodium was decreased by 3–5 mg/kg/d for elevations in serum creatinine. Of the 33 patients enrolled in the trial, 30 were evaluable for response; the overall response rate was 89% (*n* = 24) 24, including two complete responses (CR), four very good partial responses (VGPR), and twelve partial responses (PR). Six patients had a minor response (MR) and six patients had progressive disease (PD). The estimated PFS was 12 months, the median OS was 17.4 months, and the median duration of response was 13 months. Of nine patients evaluated using real-time quantitative RT-PCR of bone marrow aspirates, six demonstrated a significant decrease in Bcl-2 mRNA expression and five of these patients had a clinical response (≥ PR) [12]. In another phase II study, oblimersen sodium was combined with VAD (vincristine, adriamycin, dexamethasone) chemotherapy in 10 patients with RRMM who had received a median of four prior chemotherapy regimens, and eight of these patients (80%) had VAD refractory disease [13]. Seven patients (70%) responded to the combination of oblimersen sodium and VAD; four (40%) achieved a PR, and three (30%) achieved an MR. The median PFS of these patients was six months (range: 2–7 months). The median OS for all patients at the time of publication exceeded 12 months (range: 3–16 months). Correlative analysis revealed that oblimersen sodium downregulated Bcl-2 protein levels in peripheral blood-circulating myeloma cells, as well as B cells, T cells, and monocytes. These results suggest that oblimersen sodium, via inhibition of Bcl-2 protein synthesis, may play a role reversing resistance to chemotherapy and restoring myeloma tumor-cell sensitivity to chemotherapy with VAD [13]. While these results seemed promising, oblimersen sodium was ultimately not approved by the FDA for the treatment of RRMM or any malignancy due to lack of evidence of effectiveness in multiple phase III trials [14].

## 3. AT-101

AT-101 is an oral small molecule inhibitor, which has the ability to target both Bcl-2 and Mcl-1, and, to lesser degree, Bcl-xL and Bcl-w [15]. We observed in preclinical models of MM that, by disrupting Bcl-2 and Mcl-1 function, AT-101 potentiates the cytotoxic effects of lenalidomide and dexamethasone [16]. In human-derived chronic lymphocytic leukemia cell lines, pre-treatment of cells with AT-101 was shown to potentiate the cytotoxic effects of lenalidomide and rituximab via the potentiation of antibody-dependent cellular cytotoxicity [17]. A phase I trial evaluated the combination of AT-101, lenalidomide, and dexamethasone in patients with RRMM [18]. In this trial, 10 patients with RRMM who were previously treated with a median of two prior lines of therapy were treated with AT-101, lenalidomide, and dexamethasone until disease progression. Thirty percent of patients were lenalidomide-refractory, 20% were bortezomib-refractory and 30% were daratumumab-refractory. Only one patient had t (11;14). The median duration of the therapy was 7.5 cycles. The ORR was 44% (2 VGPR and 2 PR) and the clinical benefit rate (CBR) was 89% (2 VGPR, 2 PR, 2 MR, 2 SD). The median PFS for all patients was 8.1 months. No tumor lysis syndrome was observed and there were no grade 3/4 non-hematological AEs. ≥ Grade 3 hematological AEs were white blood cell count decrease (*n* = 3), neutropenia (*n* = 5), febrile neutropenia (*n* = 1), and thrombocytopenia (*n* = 2). Correlative analysis from patients who showed an objective response to treatment revealed a decrease in Bcl-2:Bim or Mcl-1:Noxa protein complexes, an increase in CD8+ T and NK cells, and a depletion of T and B-regulatory cells [18]. These findings support further investigation of AT-101 for the treatment of patients with RRMM.

## 4. Venetoclax

Venetoclax is a selective, orally bioavailable Bcl-2 inhibitor. In vitro data in human MM cell lines and primary MM samples that were positive for the (11;14) translocation showed a high sensitivity to venetoclax. The sensitivity to venetoclax correlated with higher ratios of Bcl-2 to Mcl-1 mRNA; higher Bcl-2 levels and lower Mcl-1 levels correlated with greater sensitivity to venetoclax [19]. The promising pre-clinical results with venetoclax led to the development of several phase I studies in RRMM. In one phase I study, 66 heavily pre-treated patients were enrolled (median of five prior lines of therapy), 30 of whom were positive for t (11;14). The patient population was highly refractory, with 70% refractory to bortezomib, 77% refractory to lenalidomide, and 53% refractory to pomalidomide. In total, 76% of patients had undergone a prior autologous stem cell transplant. Venetoclax was given daily at 300, 600, 900, or 1200 mg in the dose escalation cohorts and at 1200 mg in the safety expansion cohort. The ORR was 21% (14/66), and 15% achieved ≥ VGPR. The majority of responses (12/14 (86%)) were noted in patients with t (11;14); the ORR was 40%, with 27% of patients achieving ≥ VGPR. The most common adverse events were gastrointestinal in nature and included nausea (47%), diarrhea (36%), and vomiting (21%). The most common ≥ Grade 3 events were hematological in nature with thrombocytopenia (32%), neutropenia (27%), anemia, and leukopenia (both 23%) [20]. A subsequent phase I trial evaluated venetoclax combined with dexamethasone in t (11;14)-positive patients with RRMM. This study had a phase I portion and a phase II portion. The primary objective of the phase I portion of the trial was to assess the safety and pharmacokinetics of venetoclax–dexamethasone and the primary objective of the phase II portion of the trial was to further evaluate efficacy in addition to ORR and the ≥ VGPR rate. In the phase I portion of the trial, 20 patients who had received a median of three prior lines of therapy were enrolled. Ninety percent, 65%, and 20% had a disease that was refractory to a prior IMiD, PI, and daratumumab, respectively. The ORR was 60%, with 30% achieving ≥ VGPR. The median duration of response (DOR) was 12.4 months. ≥ Grade 3 hematological AEs that occurred in ≥10% of patients included lymphopenia (20%), neutropenia (15%), thrombocytopenia (10%), and anemia (5%). Thirty-one patients with a median of five prior lines of therapy participated in the phase II portion of the trial. In total, 87% of patients were refractory to a prior IMiD, PI, or daratumumab. The ORR was 48%, with 36% achieving ≥ VGPR. The estimated DOR at 12 months was 61%. ≥ Grade 3 hematological AEs that occurred in ≥ 10% of patients included lymphopenia (19%), neutropenia (7%), thrombocytopenia (10%), and anemia (16%) [21].

In addition to having single-agent activity, venetoclax may synergistically enhance the levels of apoptosis induced by other anti-MM cells. PIs have the ability to upregulate the BH3-only protein NOXA, an anti-apoptotic protein which binds to and inhibits the activity of Mcl-1. By upregulating the levels of NOXA, the addition of PI to venetoclax and dexamethasone may inhibit a potential source of resistance to venetoclax by lowering the levels of Mcl-1 [22]. In the phase III BELLINI trial, 291 patients were randomized to receive venetoclax, bortezomib, and dexamethasone, or bortezomib and dexamethasone in a 2:1 ratio. With a median follow-up of 18.7 months, the median PFS was 22.4 months (95% CI 15.3–not estimable) with venetoclax, bortezomib, and dexamethasone, versus 11.5 months (95% CI 9.6–15.0) with bortezomib and dexamethasone (HR 0.63; *p* = 0.010). The most common ≥ Grade 3 treatment-emergent AEs in the venetoclax, bortezomib, and dexamethasone group were neutropenia (18%), pneumonia (16%), thrombocytopenia (15%), anemia (15%), and diarrhea (15%), compared to 7% neutropenia, 9% pneumonia, 30% thrombocytopenia, 15% anemia, and 11% diarrhea in the bortezomib and dexamethasone group. Serious treatment-emergent AEs occurred in 48% of patients in the venetoclax, bortezomib, and dexamethasone group vs. 50% of patients in the bortezomib and dexamethasone group, with 4% treatment-emergent fatal infections reported in the venetoclax, bortezomib, and dexamethasone group and none reported in bortezomib and dexamethasone group. An increased proportion of overall survival events was observed in the venetoclax, bortezomib, and dexamethasone group; 21% of events occurred in the venetoclax, bortezomib, and dexamethasone group, compared with 11% in the bortezomib and dexamethasone group (HR 2.03 (95% CI 1.04–3.95); *p* = 0.034) [23]. In March of 2019, the U.S. Food and Drug Administration placed a partial clinical hold on all trials evaluating venetoclax for the treatment of RRMM based on the increased proportion of overall survival events that occurred in patients who received venetoclax in the BELLINI trial. [24]. This hold was subsequently lifted and the development of venetoclax-based clinical trials has been allowed to continue, but with a biomarker-directed approach for patients with RRMM with t (11;14) translocation only [25]. With subsequent follow-up, an OS analysis in the phase III BELLINI trial revealed that the addition of venetoclax to bortezomib and dexamethasone showed significantly improved PFS but resulted in increased mortality versus placebo in the total population (HR 1.19 (95% CI 0.80–1.77), but not in patients with t (11;14); HR 0.61 (95% CI 0.16–2.32) [26]. This further supports the role of venetoclax-only treatment for RRMM patients with t (11;14).

A phase II, dose-escalation and expansion study evaluated the combination of venetoclax, pomalidomide, and dexamethasone in patients with RRMM. Eight patients were enrolled. All patients received 400 mg of venetoclax in addition to standard doses of pomalidomide and dexamethasone; four patients experienced dose-limiting toxicities and the dose of venetoclax was not escalated to the planned 800 mg. All patients had a grade ≥ 3 AE, the majority of which were cytopenias. Five patients (63%) had a confirmed response (≥PR); the median DOR was 12.9 months, and the median PFS was 10.5 months. Due to the results of the phase III BELLINI trial, which led to a partial clinical hold of all studies evaluating the investigational use of venetoclax in RRMM and the subsequent decision to only evaluate venetoclax in patients with RRMM that harbored t (11;14), no additional patients were enrolled in this study [27]. Another phase II study evaluated the combination of venetoclax, carfilzomib, and dexamethasone in patients with RRMM with and without t (11;14). Forty-nine patients received treatment and had received a median of one (range, 1–3) prior line of therapy. The median time in the study was 27 months. The most common treatment-emergent AEs were diarrhea (65%), fatigue (47%), nausea (47%), and lymphopenia (35%). Serious AEs occurred in 26 (53%) patients. The ORR was 80% in the entire cohort of patients, 92% in patients with t(11;14) (*n* = 13), and 75% in patients without t (11;14) (*n* = 36). The ≥ CR rate was 41% and the median PFS was 22.8 months [28].

Another phase I study evaluated venetoclax with daratumumab and dexamethasone (VenDd) in patients with t (11;14) RRMM and venetoclax, daratumumab, bortezomib, and dexamethasone (VenDVd) in patients with RRMM, regardless of cytogenetics. The primary objectives included safety and ORR. In total, 48 patients were enrolled, 24 in each cohort. After a median follow-up time of 20.9 months in the VenDd group and 20.4 months in the VenDVd group, the ORR was 96% with VenDd and 92% with VenDVd, with a ≥ 75% VGPR rate in both cohorts. The 18-month PFS rate was 90.5% (95% CI, 67.0 to 97.5) with VenDd, and 66.7% (95% CI, 42.5 to 82.5) with VenDVd. Common AEs with VenDd and VenDVd included diarrhea (63% and 54%) and nausea (50% and 50%, respectively). Moreover, ≥ 3 Grade 3 AEs were observed in 88% of patients in the VenDd group and in 71% of patients in the VenDVd group [29]. Due to drug supply issues, active recruitment has been withdrawn from this trial.

The phase III, multicenter, randomized CANOVA trial (NCT03539744) compares venetoclax, pomalidomide, and dexamethasone to pomalidomide and dexamethasone in patients with t (11;14)-positive RRMM. The trial has an estimated enrollment of 244 participants and has the primary objective of comparing PFS between venetoclax, pomalidomide, and dexamethasone and pomalidomide and dexamethasone. A phase Ib/II randomized, triple-arm trial of cobimetinib, administered as a single agent and in combination with venetoclax, with or without atezolizumab, in patients with RRMM has completed accrual of 49 patients; results are awaited (NCT03312530). A summary of all the reported venetoclax-based studies in RRMM is shown in Table 2.

Most studies with venetoclax in RRMM have used 800 mg as the treatment dose. This dose appears to be well tolerated even in combination with other anti-myeloma agents such as proteasome inhibitors, immunomodulatory drugs, and monoclonal antibodies. The optimum duration of treatment with venetoclax-based therapy in RRMM remains unknown. Most clinical trials evaluating venetoclax in RRMM have treated patients until disease progression, and this appears to be the correct strategy with venetoclax use. In lymphoid malignancies, such as chronic lymphocytic leukemia, time-limited therapy with venetoclax-based regimens leads to durable remissions [30,31]; in plasma cell dyscrasias, meanwhile, time-limited therapy does not lead to durable remissions, as is evidenced in the data of venetoclax in Waldenstrom’s macroglobulinemia. In a phase II trial of relapsed and refractory Waldenstrom’s macroglobulinemia, patients were treated with venetoclax for two years and then treatment was discontinued. After a median follow-up of 33 months, 19 out of 32 patients (59%) developed disease progression, with 68% (*n* = 13) of those patients developing disease progression after completing two years of venetoclax and while not receiving therapy [32].

## 5. APG2575

APG-2575 (Lisaftoclax) is a selective and potent Bcl-2 inhibitor that effectively competes with BIM in the Bcl-2/BIM complex [33]. Six patients with RRMM were included in the first in-human, global phase I dose study that assessed the safety, pharmacokinetics, pharmacodynamics, efficacy, and maximum tolerated dose (MTD)/recommended phase 2 dose (RP2D) of single agent APG2575 in relapsed/refractory hematologic malignancies. APG2575 doses ranged from 20 to 1200 mg, and no DLTs were observed at the maximum assessed dose of 1200 mg. Patients received a median of seven cycles of treatment (range: 3–20). Twelve of fourteen evaluable patients with relapsed/refractory chronic lymphocytic leukemia/small lymphocytic lymphoma (CLL/SLL) achieved a PR, resulting in an impressive ORR of 85.7%. The median time to response was three cycles (range 2–7). No evidence of tumor lysis syndrome was noted. Treatment-related AEs of any grade that occurred in > 10% of patients were mainly gastrointestinal and hematologic in nature; these AEs included diarrhea (17.1%), nausea (11.4%), neutropenia (22.9%), anemia (17.1%), and fatigue (28.6%). ≥ Grade 3 treatment-related AEs were mainly hematological in nature: neutropenia (14.3%), and thrombocytopenia, leukopenia, lymphopenia, fatigue, and nausea (2.9% of patients each) [34]. This treatment’s efficacy in patients with RRMM has not yet been reported. Several trials are underway that evaluate the efficacy of APG2575 in combination with other anti-MM agents in RRMM (NCT04942067, NCT04674514).

## 6. S-55746

S-55746 is an orally bioavailable and potent inhibitor of Bcl-2; it has no significant binding to Mcl-1 and poor affinity for Bcl-xL [35]. A phase I dose-escalation study of S-55746 in patients with relapsed/refractory CLL and B-cell non-Hodgkin lymphomas, including patients with RRMM (NCT02920697), has completed accrual; results are awaited.

## 7. BGB-11417

BGB-11417 is a potent and selective Bcl-2 inhibitor that has exhibited superior anti-tumor activity compared to venetoclax in human acute lymphoblastic leukemia, mantle cell lymphoma, and diffuse large B-cell lymphoma mouse xenograft models [36]. BGB-11417–101 is a phase 1 dose-escalation and expansion study that enrolled patients with CLL/SLL or other non-Hodgkin lymphomas (NHL). Patients were treated with BGB-11417 as a monotherapy or in combination with the Bruton’s tyrosine kinase inhibitor zanubrutinib. In the dose escalation portion of the study, patients were enrolled in one of five potential dose levels of BGB-11417: 40, 80, 160, 320, or 640 mg once daily. As of data cutoff (24 May 2021), 14 patients (NHL: *n* = 11; CLL/SLL: *n* = 3) who had received a median of two prior lines of therapy (range, 1–4) were enrolled in the study and were treated with BGB-11417 monotherapy. There were no DLTs observed in patients with NHL receiving BGB-11417 monotherapy (*n* = 11) up to the 160 mg dose level. A total of five patients discontinued treatment (all NHL) due to disease progression (*n* = 4; 2 at 40 mg, 2 at 80 mg) or lack of efficacy (*n* = 1 at 40 mg). No patients discontinued due to AEs. Grade ≥ 3 AEs that occurred included an AST increase in one patient (7.1%), neutropenia in three patients (21.4%), hypokalemia in one patient (7.1%), and pyrexia in one patient (7.1%) [37]. Other clinical trials with BGB-11417 alone or in combination with other agents are underway in patients with RRMM. A phase Ib/II clinical trial evaluating the safety and efficacy of BGB-11417 in combination with dexamethasone and/or carfilzomib/dexamethasone in patients with RRMM and t (11;14) is currently enrolling patients (NCT04973605).

## 8. AZD-0466

Previous pre-clinical and clinical work with ABT-737 demonstrated that ABT-737 has nanomolar affinity for the BH3 binding site of Bcl-2, Bcl-xL, and Bcl-w, and is capable of inducing apoptosis in a range of malignancies in vitro [38]. ABT-737 was modified so that it would be orally bioavailable, resulting in ABT-263 (navitoclax). Clinical development of navitoclax was ultimately halted due to significant thrombocytopenia resulting from the Bcl-xL-dependent apoptosis of platelets [39]. Targeting Bcl-xL in addition to Bcl-2 is a viable therapeutic strategy, as increased levels of Bcl-XL have been observed in a myeloma xenograft model that was resistant to venetoclax [40]. The Bcl-xL/Bcl-2 inhibitor, AZD-4320, was designed for once-weekly intravenous delivery rather than oral administration. Transient thrombocytopenia was observed, with platelets recovering within 48-72 h, but significant, dose-limiting cardiac toxicity was noted with AZD-4320 [41]. To overcome this toxicity, AstraZeneca designed a novel compound, whereby AZD-4320 is conjugated via a hydrolytically labile linker to the DEP^®^ dendrimer nanomedicine platform [42]. The novel compound, AZD-0466, diminishes the cardiovascular toxicities observed with AZD-4320, and has shown in vitro and in vivo efficacy in a range of hematologic malignancies [42]. A phase I trial evaluating the safety and tolerability of AZD-0466 in patients with advanced hematologic and solid malignancies has completed accrual (NCT04214093). A phase I/II trial evaluating the safety and efficacy of AZD-0466 monotherapy as well as in combination in patients with relapsed hematologic malignancies, including RRMM, is currently recruiting patients (NCT04865419).

## 9. MCL-1 Inhibition

Mcl-1 expression is crucial for plasma cell survival. Pre-clinical studies have shown that loss of Mcl-1 leads to the rapid disappearance of plasma cells [43]. Mcl-1 is also critical for the survival of human MM cells in vitro, as higher levels of Mcl-1 in malignant plasma cells from patients with MM have been correlated with relapse and shorter OS [44] Furthermore, the upregulation of Mcl-1 has been implicated in venetoclax resistance in MM [45]. Bone marrow samples from eight patients with MM pre-venetoclax treatment and at the time of disease progression while on venetoclax revealed a gain in the Mcl-1 mRNA transcript and downregulation of Bcl-2 at the time of relapse using single-cell RNA profiling. Cell trajectory analysis and the pseudotime ordering of cells (Monocle) revealed the emergence of a highly proliferative clone and of an Mcl-1-dependent clone as the disease evolved from its original Bcl-2-dependent cluster at pseudotime (t0) [46]. In MM cell lines, cells became highly sensitized to venetoclax when Mcl-1 was silenced using small interfering RNA, indicating that Mcl-1 plays a crucial role in conferring resistance to venetoclax [19]. Inhibiting Mcl-1 transcription using the CDK9 inhibitor flavopiridol in MM cell lines resulted in increased sensitivity of the MM to venetoclax therapy [47]. The aforementioned data makes Mcl-1 an attractive therapeutic target for the treatment of RRMM. Several trials are underway with novel agents that inhibit either Mcl-1 or Mcl-1 and Bcl-2 simultaneously in RRMM.

## 10. AMG-176 and AMG-397

AMG-176 is a selective Mcl-1 inhibitor that disrupts the interactions of the Mcl-1-Bak complex and has been reported to induce rapid apoptosis in different hematologic malignancies [48,49]. Preclinical studies have demonstrated the favorable safety profile and efficacy of oral AMG-176 in MM xenograft models [49]. Although AMG-176 was administered orally in preclinical studies, intravenous infusion is being used in clinical studies. Intravenous administration of AMG-176 has been evaluated in a phase I clinical trial in patients with RRMM (NCT02675452), and preliminary safety and efficacy have been reported [50]. At the data cutoff date (15 March 2019), 26 patients with RRMM, with a median age of 63.5 years and a median of five prior lines of therapy, received AMG-176. Eleven patients achieved stable disease as the best overall response. Patients received a median of two (range 1–8) cycles of AMG-176. Most patients discontinued treatment due to progressive disease (*n* = 22, 85%) Treatment-emergent adverse events (TEAEs) of any grade occurred in 25 patients (96%). Common TEAEs were mostly hematological and gastrointestinal in nature; they included neutropenia (*n* = 10 (38%)), nausea (*n* = 8 (31%)), diarrhea (*n* = 7 (27%)), and anemia (*n* = 6 (23%)) [50]. Because of the intravenous administration required for AMG-176, Amgen has developed AMG-397, a potent, selective, and orally bioavailable Mcl-1 inhibitor. Refinements in the potency and pharmacokinetics of the AMG-176 molecule led to the development of AMG-397. In cells, AMG-397 potently disrupted the interaction between Mcl-1 and Bim. Treatment of OPM2 cells, an Mcl-1 dependent MM cell line, induced clear increases in Caspase-3/7 activity within one hour. In vivo, oral AMG-397 demonstrated rapid and sustained dose-dependent increases in activated BAK, cleaved Caspase-3, and cleaved PARP in established OPM2 xenografts. Dosing schedules of once or twice weekly at 25 or 50 mg/kg of AMG-397 exhibited significant tumor regressions in mice bearing OPM2 xenografts, with nine out of ten mice tumor-free at the end of the study in both 50 mg/kg groups [51]. AMG-397 has been placed on a clinical hold by the U.S. FDA, based on a safety signal of cardiac toxicity. As such, Amgen has also voluntarily halted enrollment in the phase I trial NCT02675452 with AMG-176.

## 11. S-64315

S-63845 is a selective BH3 mimetic Mcl-1 inhibitor that causes apoptosis of various Mcl-1 dependent hematologic malignancies by activating the Bax/Bak dependent mitochondrial apoptotic pathway [52]. S-64315 binds to the BH3-binding groove of Mcl-1 and does not bind to Bcl-2 or Bcl-xL. IV infusion of S-63845 once per day for five consecutive days resulted in 100% tumor regression in MM subcutaneous xenograft models [52,53]. The combination of S63845 and venetoclax was tested in vitro, ex vivo, and in vivo in malignant plasma cells from patients with MM [53]. In vitro data revealed that the combination of S-63845+venetoclax increased apoptosis and decreased cell viability in malignant plasma cells, and the triplet combination of S-63845+venetoclax+dexamethasone showed even higher levels of apoptosis. The combination of S63845 and venetoclax was also tested ex vivo in malignant plasma cells isolated from eight MM patients. In five out of the eight MM patients, the combination of S63845 and venetoclax enhanced the levels of apoptosis compared to each agent alone. In an in vivo disseminated mouse model of MM, the triplet combination of S-63845+venetoclax+dexamethasone induced an approximately 30-day delay in tumor growth, compared with the control group. This combination appeared to have good tolerability, without significant losses in body weight or other signs of toxicity [54]. In another study of human MM cell lines, the combination of S-63845 and venetoclax enhanced the sensitivity of MM cells to venetoclax and overcame resistance to venetoclax [55]. Novartis has created another S-derivative called S-64315 or MIK665. A phase I study, which characterized the safety, tolerability, and pharmacokinetics of intravenously administered MIK665 in patients with relapsed/refractory MM or lymphoma has completed accrual and the results are forthcoming (NCT02992483). A phase Ib study of S-65487 (a Bcl-2 inhibitor) in combination with MIK665 in patients with relapsed/refractory MM, acute myeloid leukemia, and non-Hodgkin lymphoma is actively recruiting participants (NCT04702425).

## 12. PRT-1419

PRT-1419 is a selective oral inhibitor of Mcl-1 that disrupts the formation of the Mcl-1-BIM complex with low nanomolar potency; it has negligible binding to Bcl-2 and Bcl-xL [56]. PRT-1419 demonstrated good oral bioavailability and favorable pharmacokinetic properties in vivo. In xenograft models of MM, oral administration of PRT-1419 demonstrated anti-tumor activity, with complete tumor regressions observed at well-tolerated doses [56]. PRT-1419 is currently being evaluated in a phase 1 dose-escalation study in patients with relapsed/refractory hematologic malignancies, including MM (NCT04543305).

## 13. AZD-5991

AZD-5991 is a macrocyclic molecule with high selectivity and affinity for Mcl-1, due to the fact that it binds directly to Mcl-1; it induces rapid apoptosis in cancer cells, most notably MM and acute myeloid leukemia, via activation of the Bak-dependent mitochondrial apoptotic pathway [57]. In MM xenograft models, AZD-5991 exhibited potent activity, showing 100% tumor regression in four out of four mice after a single IV dose. A sub-efficacious dose of AZD-5991 in combination with bortezomib induced 88% tumor regression in an MM xenograft model [57]. Confirming these in vitro results, the combination of AZD-5991 and venetoclax in OCI-AML3 subcutaneous mouse models of acute myeloid leukemia (AML) (an AML model that is inherently resistant to AZD-5991 and venetoclax monotherapies) led to tumor regression in eight out of eight mice [55]. There was a phase I study of AZD-5991 in relapsed/refractory hematologic malignancies, and a phase II portion of AZD-5991 plus venetoclax in relapsed/refractory AML (NCT03218683). A US clinical hold has been placed on this clinical trial, however, due to asymptomatic elevations in cardiovascular laboratory parameters that were noted in an AML patient treated with AZD-5991 and venetoclax [58].

Further safety data need to be generated to understand the safety profile of Mcl-1 inhibition in RRMM. In preclinical studies, conditional Mcl-1 knockout in mice revealed an important role for Mcl-1 in the survival of cardiac myocytes [59,60]. Mice in which Mcl-1 was genetically deleted from the heart rapidly developed cardiomyopathy and died. This cardiac toxicity arose not just because of the apoptosis of cardiac myocytes, but also due to mitochondrial dysfunction in the myocytes. Therefore, further evaluation is necessary, both because of these early signs of cardiac toxicity with several Mcl-1 inhibitors in RRMM, as well as when combining Mcl-1 inhibition with Bcl-2 inhibition, and because of the potential for exacerbation of the known cardiovascular side effects of several anti-MM agents, such as proteasome inhibitors.

## 14. Conclusions

Several studies have shown that MM becomes increasingly dependent on the Bcl-2-family proteins for survival during tumorigenesis, which makes targeting these proteins a rational therapeutic approach. Most of the Bcl-2-targeting agents are in the early stages of clinical development; their optimal usage, as well as the question of how to combine these agents with other myeloma-targeting drugs, is being actively investigated. Importantly, in vitro studies have shown heterogeneity with regards to which anti-apoptotic Bcl-2-family proteins MM cells are dependent on, which further complicates the ideal utilization of Bcl-2-family inhibitors in RRMM. Venetoclax is the Bcl-2 inhibitor that is farthest along in clinical development. It has shown clinical activity as a single agent and when combined with dexamethasone, bortezomib, carfilzomib, pomalidomide, and daratumumab. The phase III BELLINI trial showed superior PFS and ORR for patients with t (11;14) positive RRMM, but an increased death signal for patients without t (11;14) who were receiving venetoclax, bortezomib, and dexamethasone; this suggests that venetoclax is perhaps best utilized in patients with t (11;14)-positive RRMM. Several other Bcl-2-specific inhibitors and a Bcl-2/xL inhibitor are currently in early phase trials, as are Mcl-1 inhibitors. While the combination of dual Bcl-2 and Mcl-1 inhibition makes biological sense, the increased cardiac toxicity signal seen with this combination, which arises as a result of cardiac myocytes’ dependence on Mcl-1, warrants more careful examination of this therapeutic combination in RRMM. The future appears promising for small molecule inhibitors that target the Bcl-2-family proteins, and the results of the multitude of clinical trials underway with these agents are eagerly awaited.

## Figures and Tables

**Table 1:** Ongoing clinical trials with Bcl-2-family inhibitors in relapsed refractory multiple myeloma.

ClinicalTrials.Gov Identifier	Phase	Estimated or Actual Enrollment	Arm(s)	Primary Endpoints
**BCL-2 Inhibitors**				
**Venetoclax**				
NCT02899052	2	120	Carfilzomib + venetoclax and dexamethasone	Safety, ORR, ≥VGPR rate, ≥CR rate
NCT03539744	3	244	Venetoclax + pomalidomide and dexamethasone vs. pomalidomide and dexamethasone	PFS
**APG-2575**				
NCT04942067	1/2	108	APG-2575 + pomalidomide and dexamethasone and APG-2575 + daratumumab, lenalidomide and dexamethasone	DLT rate, MTD/RP2D
NCT04674514	1/2	48	APG-2575 + lenalidomide and dexamethasone	DLT rate, MTD/RP2D
**S-55746**				
NCT02920697	1	65	S-557646	Safety, MTD
**BGB-11417**				
NCT04973605	1/2	146	BGB-11417 and BGB-11417 + dexamethasone and BGB-11417 + carfilzomib and dexamethasone	Part 1: safety, DLT Part 2: ORR, ≥VGPR rate, ≥CR rate
**BCL-2 and BCL-XL Inhibitors**				
**AZD-0466**				
NCT04214093	1	9	AZD-0466	Safety, MTD
NCT04865419	1/2	141	AZD-0466	Safety
**MCL-1 Inhibitors**				
**AMG-176**				
NCT02675452	1	175	AMG-176	Safety
**S-64315**				
NCT02992483	1	31	S-64315	Safety
**PRT-1419**				
NCT04543305	1	36	PRT-1419	Safety, MTD/RP2D
**AZD-5991**				
NCT03218683	1	144	AZD-5991	Safety, MTD
**BCL-2 and MCL-1 Inhibitors**				
NCT04702425	1	170	S64315 + S65487	Safety

CR: complete response, DLT: dose limiting toxicity, MTD: maximum tolerated dose, ORR: overall response rate, PFS: progression-free survival, RP2D: recommended phase 2 dose, VGPR: very good partial response.

**Table 2:** Studies with venetoclax-based regimens in relapsed/refractory multiple myeloma.

ClinicalTrials.Gov Identifier	Phase/# of Patients	Regimen	Median Prior Lines (Range)	Maximum Tolerated Dose of Venetoclax	Refractoriness to Lenalidomide and Bortezomib	Cytogenetics	ORR	Median PFS	Most Common Grade 3/4 Adverse Events
NCT01794520	I/66	Venetoclax	5 (1–15)	1200 mg	Len: 77% Bor: 70%	t (11;14): 45% High Risk:27%	21% 40% in t (11;14)	NR	thrombocytopenia (32%), neutropenia (27%), anemia (23%), and leukopenia (23%)
NCT01794520	I/20	Venetoclax and dexamethasone	3 (1–7)	800 mg	Len: 85% Bor: 55%	t (11;14): 100% High Risk: NR	65%	NR	lymphopenia (15), hypophosphatemia (15%), hyperuricemia (10%) and laboratory tumor lysis syndrome (10%)
NCT02755597	III/291	Venetoclax, bortezomib, and dexamethasone	53% received 2–3 prior lines	800 mg	Len: 20% Bor: None	t (11;14): 10% High Risk:16%	82%; 90% in t(11;14)	22.4 months	neutropenia (18%), pneumonia (16%), thrombocytopenia (15%), anemia (15%), and diarrhea (15%)
NCT03567616	II/8	Venetoclax, pomalidomide, and dexamethasone	1.5 (1–5)	800 mg	Len: 75% Bor: NR	t (11;14): 37.5% High Risk: 62.5%	62.5%; 66.7% in t(11;14)	10.5 months	neutropenia (75%), leukopenia (38%), lymphocyte count decreased (25%), neutrophil count decreased (25%)
NCT02899052	II/49	Venetoclax, carfilzomib, and dexamethasone	1 (1–3)	800 mg	Len: 67% Bor: 47%	t (11;14): 27% High Risk: 27%	80%; 92% in t(11;14)	22.8 months	hypertension (16%), pneumonia (12%), lymphopenia (31%), leukopenia (12%), and neutropenia (12%)
NCT03314181	I/48	Venetoclax +/− bortezomib, daratumumab, and dexamethasone	−Bor:2.5 (1–8) +Bor:1 (1–3)	800 mg	Len: NR Bor: NR	t (11;14): 62.5% High Risk: 10%	−Bor: 96% +Bor: 92%	−Bor: 90.5% at 18 m +Bor: 66.7% at 18 m	−Bor: hypertension (17%), neutropenia (21%), lymphopenia (8%) +Bor: insomnia (25%), thrombocytopenia (17%), lymphopenia (13%)

NR: not reported, Len: lenalidomide, Bor: bortezomib.
